# Nearest Correlation-Based Input Variable Weighting for Soft-Sensor Design

**DOI:** 10.3389/fchem.2018.00171

**Published:** 2018-05-22

**Authors:** Koichi Fujiwara, Manabu Kano

**Affiliations:** Department of Systems Science, Kyoto University, Kyoto, Japan

**Keywords:** soft-sensor, calibration model, variable weighting, partial least squares, near infrared spectroscopy

## Abstract

In recent years, soft-sensors have been widely used for estimating product quality or other important variables when online analyzers are not available. In order to construct a highly accurate soft-sensor, appropriate data preprocessing is required. In particular, the selection of input variables or input features is one of the most important techniques for improving estimation performance. Fujiwara et al. proposed a variable selection method, in which variables are clustered into variable groups based on the correlation between variables by nearest correlation spectral clustering (NCSC), and each variable group is examined as to whether or not it should be used as input variables. This method is called NCSC-based variable selection (NCSC-VS). However, these NCSC-based methods have a lot of parameters to be tuned, and their joint optimization is burdensome. The present work proposes an effective input variable weighting method to be used instead of variable selection to conserve labor required for parameter tuning. The proposed method, referred to herein as NC-based variable weighting (NCVW), searches input variables that have the correlation with the output variable by using the NC method and calculates the correlation similarity between the input variables and output variable. The input variables are weighted based on the calculated correlation similarities, and the weighted input variables are used for model construction. There is only one parameter in the proposed NCVW since the NC method has one tuning parameter. Thus, it is easy for NCVW to develop a soft-sensor. The usefulness of the proposed NCVW is demonstrated through an application to calibration model design in a pharmaceutical process.

## 1. Introduction

It is important in terms of process safety and quality control to estimate product quality or other process variables, particularly when online analyzers are not available. Soft-sensors are mathematical models for estimating variables that are difficult to measure by hard sensors in real-time from other variables that are easy to measure. They have been used in various industries, for example, measurement of product composition at distillation columns in chemical processes, silicon wafer surface flatness in semiconductor processes, and active ingredient content of drugs in pharmaceutical processes. There are three methodologies for constructing soft-sensors: (i) first-principal modeling based on physicochemical knowledge of processes, (ii) statistical modeling based on process data, and (iii) a combination of the two. These methodologies also are called white-box, black-box, and gray-box modeling, respectively (Ahmad et al., 2014). In particular, statistical modeling has attracted wide attention due to recent advances in machine learning. Although we can utilize various machine learning techniques for soft-sensor development, partial least squares (PLS) is still widely used in chemometrics as well as soft-sensor design. This is because it is possible to construct an accurate linear regression model even when the multicollinearity problem occurs (Wold et al., 2001; Kano and Ogawa, 2010; Kano and Fujiwara, 2013).

One of the major issues in developing a precise soft-sensor is input variable selection. Although soft-sensors are well-fitted to modeling data when numerous variables are used as the input, their performance may deteriorate when unimportant variables are used for estimation. In particular, input variable selection is a key when a calibration model is constructed from Near-infrared spectroscopy (NIRS) which is a powerful online measurement technology due to its short measuring time and non-invasiveness (Roggo et al., 2007; Miyano et al., 2014). The number of measured wavelengths of an NIR spectrum is usually more than 100.

If all of the possible variable combinations are tested, the computational load increases exponentially as the candidate variables increase. Appropriate variables must be selected in a systematic manner, which is referred to as input variable selection in soft-sensors, and feature selection in machine learning. A technique for input variable selection should be developed for improving the efficiency of soft-sensor design (Andersen and Bro, 2010; Mehmood et al., 2012).

In linear regression, stepwise and least absolute shrinkage and selection operator (Lasso) are widely used as input variable selection methods (Hocking, 1976; Tibshirani, 1996). In addition, PLS-Beta and variable influence on projection (VIP) are available for selecting input variables of PLS (Kubinyi, 1993).

Methods of selecting variables on the basis of correlation have been proposed because the correlation between variables should be considered when building a good regression model (Fujiwara et al., 2009). In correlation-based variable selection methods, variable groups are constructed according to the correlation, some of which are selected as the input variables. Nearest correlation spectral clustering (NCSC) (Fujiwara et al., 2010, 2011) is used for variable grouping. In NCSC-based variable selection (NCSC-VS), variable groups are constructed by NCSC, and it is examined whether or not they should be used as the input variables according to their contribution to the estimates (Fujiwara et al., 2012b). In addition, NCSC-based group Lasso (NCSC-GL) uses group Lasso (Yuan and Lin, 2006; Bach, 2008) for variable group selection after NCSC (Fujiwara and Kano, 2015). Although both NCSC-VS and NCSC-GL can build highly-accurate soft-sensors, tuning their parameters is complicated and time-consuming because they have multiple parameters to be tuned. Therefore, the number of their tuning parameters should be reduced for efficient variable selection.

Another approach is input variable weighting or input variable scaling, which multiplies each input variable by weights according to its importance from the viewpoint of estimation (Kim et al., 2014). The present work proposes an effective input variable weighting method to replace variable selection in order to conserve labor required for parameter tuning. The proposed method, referred to herein as NC-based variable weighting (NCVW), searches input variables that have the correlation with the output variable by using the NC method and calculates the correlation similarity between each input variable and the output variable. The input variables are weighted based on the calculated correlation similarities, and the weighted input variables are used for modeling. Since there is only one parameter in the proposed NCVW, an efficient soft-sensor design is realized. In this work, the usefulness of the proposed NCVW is demonstrated through application to calibration model design for estimating active pharmaceutical ingredient (API) content.

This paper is organized as follows. Section 2 introduces conventional variable selection methods for PLS modeling, and NCVW is proposed in section 3. Section 4 reports on application results of the proposed method to pharmaceutical data. The conclusion and future work are described in section 5.

## 2. Conventional methods

This section introduces PLS and conventional input variable selection methods.

### 2.1. PLS

PLS is a widely used linear regression method in chemometrics as well as soft-sensor design. Given an input data matrix ***X*** ∈ℜ^*N*×*M*^ whose *n*th row is the *n*th input sample $x_{n}\in {ℜ}^{M}$ and an output data vector ***y*** ∈ℜ^*N*^ whose *n*th element is the *n*th output sample *y*_*n*_ ∈ℜ, ***X*** and ***y*** are mean-centered and appropriately scaled. The input ***X*** ∈ℜ^*N*×*M*^ and the output ***y*** ∈ℜ^*N*^ are broken down as follows:

(1) $$\begin{array}{rcl} X & = & {TP}^{T}+E \end{array}$$

(2) $$\begin{array}{rcl} y & = & Tb+f \end{array}$$

where ***T*** ∈ℜ^*N*×*K*^ is the latent variable matrix, whose columns are the latent variable $t_{k}\in {ℜ}^{N}\;\left(k=1,\cdots \;,K\right)$, ***P*** ∈ℜ^*M*×*K*^ is the loading matrix of ***X*** whose columns are the loading vectors $p_{k}\in {ℜ}^{M}$, and $b={\left[b_{1},\cdots \;,b_{K}\right]}^{T}$ is the regression coefficient vector of ***y***. *K* denotes the number of adopted latent variables. ***E*** ∈ℜ^*N*×*M*^ and ***f*** ∈ℜ^*N*^ are errors.

A PLS model can be constructed by the non-linear iterative partial least squares (NIPALS) algorithm. Let the first to *k*th latent variables be ***t***_1_, ⋯ , ***t***_*k*_, the loading vectors be ***p***_1_, ⋯ , ***p***_*k*_ and the loading be *b*_1_, ⋯ , *b*_*k*_. The (*k*+1)th residual input and output are as follows:

(3) $$\begin{array}{rcl} X_{k+1} & = & X_{k}-t_{r}p_{k}^{T} \end{array}$$

(4) $$\begin{array}{rcl} y_{k+1} & = & y_{k}-b_{k}t_{k}. \end{array}$$

***t***_*k*_ is a linear combination of the columns of ***X***_*k*_, that is, ***t***_*k*_ = ***X***_*k*_***w***_*k*_ where $w_{k}\in {ℜ}^{M}$ is the *k*th weighting vector. ***w***_*k*_ is the eigenvector corresponding the maximum eigenvalue of the following eigenvalue problem:

(5) $$\begin{array}{r} X_{k-1}^{T}y_{k-1}^{T}y_{k-1}X_{k-1}w_{k}=\lambda w_{k} \end{array}$$

where λ is an eigenvalue. The *k*th loading vector ***p***_*k*_ and the *k*th loading *b*_*k*_ are $p_{k}=X_{k}^{T}t_{k}/t_{k}^{T}t_{k}$ and $b_{k}=y_{k}^{T}t_{k}/t_{k}^{T}b_{k}$. This procedure is repeated until the number of adopted latent variables *K* is achieved; *K* can be determined by cross-validation.

### 2.2. PLS-beta

PLS-Beta translates a PLS model, Equations (1, 2), into a multiple linear regression (MLR) model and selects input variables based on the magnitude of its regression coefficients (Kubinyi, 1993). The translated model is expressed as

(6) $$\begin{array}{r} \hat{y}=T{\left(T^{T}T\right)}^{-1}Ty={X\beta }_{pls} \end{array}$$

where $\beta_{pls}=W{\left(P^{T}W\right)}^{-1}{\left(T^{T}T\right)}^{-1}y$, and $W=\left[w_{1},\cdots \;,w_{K}\right]\in {ℜ}^{M\times K}$. The evaluation index of PLS-Beta ν is defined as

(7) $$\begin{array}{r} \nu =\frac{\left||\beta_{select}|\right|}{\left||\beta_{pls}|\right|}\;\left(0<\nu \leq 1\right) \end{array}$$

where **β**_*select*_ is the regression coefficient vector of the selected input variables. We select individual input variables in descending order of the magnitude of **β**_*pls*_ until ν achieves a predefined threshold.

### 2.3. Variable influence on projection (VIP)

The VIP evaluates the contribution of each input variable to the output (Kubinyi, 1993). The VIP score of the *j*th input variable is

(8) $$\begin{array}{r} V_{j}=\sqrt{M\overset{K}{\underset{k=1}{\sum }}(w_{jk}^{2}b_{k}^{2}\left(t_{k}^{T}t_{k}\right)/||w_{k}|{|}^{2})/\overset{K}{\underset{k=1}{\sum }}b_{k}^{2}\left(t_{k}^{T}t_{k}\right)} \end{array}$$

where *w*_*jk*_ is the *j*th element of ***w***_*k*_. Variables satisfying *V*_*j*_ > η (>0) are selected.

### 2.4. Stepwise

Stepwise is an input variable selection method for the MLR model based on a statistical test which checks whether or not the true value of the regression coefficient of a newly added candidate variable is zero (Hocking, 1976).

### 2.5. Least absolute shrinkage and selection operator (lasso)

Lasso is least squares with *L*_1_ regularization so that some regression coefficients approach zero (Tibshirani, 1996). The objective function of Lasso is as follows:

(9) $$\begin{array}{r} \beta_{lasso}={\arg\text{ min}}_{\beta }\;\left(||y-X\beta |{|}_{2}^{2}+\lambda ||\beta |{|}_{1}\right),\;\lambda \;\left(>0\right) \end{array}$$

Least angle regression (LARS) solves the problem of Equation (9) efficiently (Efron et al., 2004).

## 3. Nearest correlation based variable weighting (NCVW)

The present work proposes a new method for weighting input variables for PLS modeling to be used instead of variable selection. Since the proposed method uses the nearest correlation (NC) method for calculating correlation-based variable weights, this section explains the NC method and variable selection methods based on the NC method before the proposed method is described.

### 3.1. NC method

The NC method was originally developed as an unsupervised learning technique for detecting samples whose correlation is similar to the query (Fujiwara et al., 2012a). The procedure of the NC method is described in Algorithm 1.

**Table:** Algorithm 1 Nearest correlation (NC) method

1: Prepare ***x***_*n*_(*n* = 1, ⋯*N*) and ***x***_*q*_.
2: Set γ.
3: **for all** *n* = 1, 2, ⋯ , *N* (*n*≠*q*) **do**
4: $x_{n}^{\prime }=x_{n}-x_{q}$.
5: **end for**
6: **for all** *k*, *l* (*k*≠*l*) **do**
7: Calculate $C_{k,l}^{\prime }$ from $x_{k}^{\prime }$ and $x_{l}^{\prime }$.
8: **if** $|C_{k,l}^{\prime }|\geq \gamma$ **then**
9: Output ***x***_*k*_ and ***x***_*l*_ as similar samples to ***x***_*q*_
10: **end if**
11: **end for**

**Figure 1:**
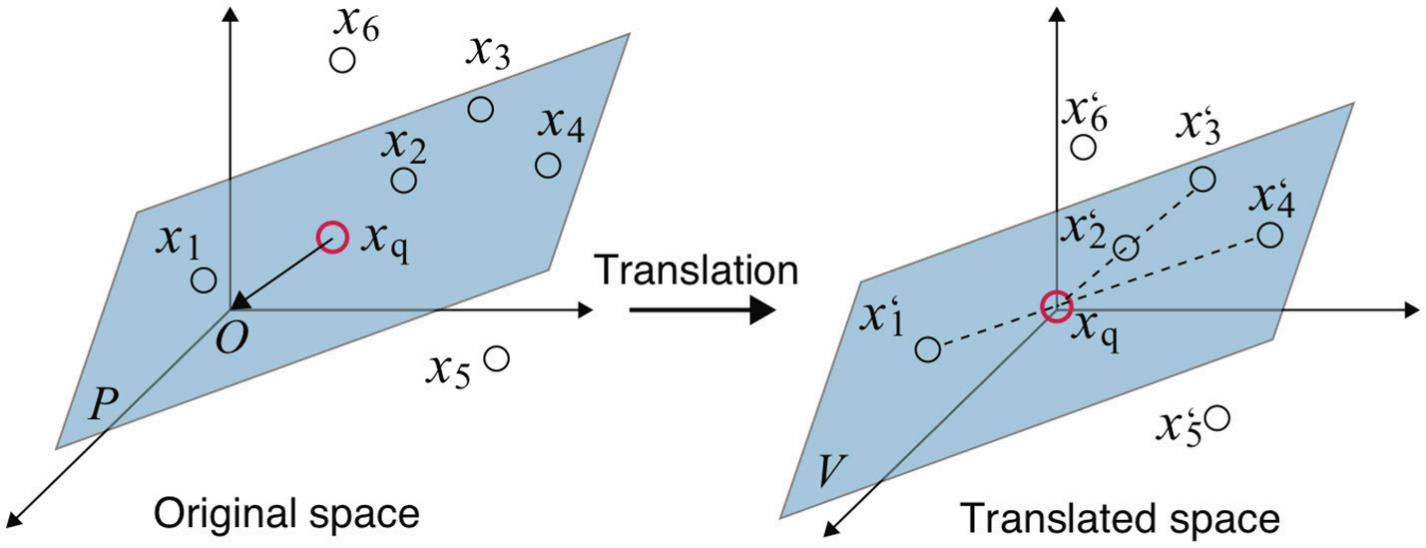
Example of the NC method: A red circle is the query ***x***_*q*_. Dashed lines denote pairs of samples, whose correlation is similar to ***x***_*q*_ (left) (Fujiwara et al., 2010, 2012a,b).

The concept of Algorithm 1 is explained through a simple example. In Figure 1 (left), there are seven samples ***x***_*q*_, ***x***_1_, ⋯ , ***x***_6_, of which five ***x***_*q*_ and ***x***_1_, ⋯ , ***x***_4_ are on the same plane *P*. That is, plane *P* expresses the hidden correlation between the five samples and ***x***_5_ and ***x***_6_ have a different correlation. The aim of the NC method here is to detect samples whose correlation is similar to the query ***x***_*q*_, that is, to detect ***x***_1_, ⋯ , ***x***_4_ on *P*.

In steps 3–5, the entire space is translated so that ***x***_*q*_ becomes the origin by subtracting ***x***_*q*_ from all other samples ***x***_*n*_ as shown in Figure 1 (right). The translated plane *P* becomes the linear subspace *V* since it contains the origin.

Draw lines connecting each sample and the origin, and check whether another sample is on the line in steps 6–8. In this example, pairs ***x***_1_-***x***_4_ and ***x***_2_-***x***_3_ satisfy such a relationship, and ***x***_5_ and ***x***_6_, which are not on *V*, cannot make pairs. At this time, the correlation coefficients of these pairs must be 1 or −1. Thus, the pairs whose correlation coefficients are ±1 are thought to have a correlation similar to ***x***_*q*_. The threshold of the correlation coefficient γ (0 < γ ≤ 1) is used for constraint relaxation. Steps 6–8 correspond to the above procedure.

Finally, the pairs whose correlations are similar to the query ***x***_*q*_ are output in step 9.

### 3.2. NCSC

NCSC was originally proposed for sample clustering based on correlation between variables (Fujiwara et al., 2010, 2011), in which the NC method and spectral clustering (SC) (Ding et al., 2001; Ng et al., 2002) are integrated. SC is a graph theory-based clustering method, which can partition a weighted graph, whose weights express affinities between nodes, into subgraphs by cutting some of their arcs. In NCSC, the NC method is used for building an affinity graph expressing the correlation-based similarities between samples, and SC partitions the graph constructed by the NC method.

Algorithm 2 shows an affinity matrix construction procedure in NCSC. Steps 6–13 correspond to the NC method, and the weighted graph constructed by the NC method is expressed as an affinity matrix ***S***. Although some SC algorithms have been proposed, the max-min cut (Mcut) algorithm (Ding et al., 2001) or its extended method (Ng et al., 2002) is used herein.

**Table:** Algorithm 2 Affinity matrix construction

1: Set γ and *J*.
2: $S\in {ℜ}^{N\times N}\leftarrow O_{N,N}$.
3: *L* = 1.
4: **for** *L* = 1 to *N* **do**
5: $S_{L}\in {ℜ}^{N\times N}\leftarrow O_{N,N}$.
6: **for all** *n* = 1, 2, ⋯ , *N* (*n*≠*L*) **do**
7: $x_{n}^{\prime }=x_{n}-x_{L}$.
8: **end for**
9: **for all** *k*, *l* (*k*≠*l*) **do**
10: Calculate $C_{k,l}^{\prime }$ from $x_{k}^{\prime }$ and $x_{l}^{\prime }$.
11: **if** $|C_{k,l}^{\prime }|\geq \gamma$ **then**
12: (_***S***_*L*_)*k, l*_ = (_***S***_*L*_)*l, k*_ = 1.
13: **end if**
14: **end for**
15: ***S*** = ***S***+***S***_*L*_.
16: **end for**

NCSC has two parameters: the threshold in the NC method γ and the number of clusters partitioned by SC, *J*. Previous studies have suggested the default value of γ to be 0.99 (Fujiwara et al., 2010, 2011), and that *J* needs to be determined by trial and error.

### 3.3. NCSC-VS and NCSC-GL

NCSC has been utilized for variable selection in soft-sensor design. In these methods, multiple variable groups are constructed by NCSC, of which some are selected as the input variables of a soft-sensor. NCSC classifies variables into *J* variable groups $v_{j}=\left\{x_{m}\;|\;m\subset V_{j}\right\}\;\left(j=1,\cdots \;,J\right)$, where $V_{j}$ is the subset of variable indexes and $V=\cup V_{j}$. An affinity matrix is derived from the transposed input variable matrix ***X***^*T*^ by the NC method for variable grouping.

NCSC-VS evaluates each variable group as to whether or not its members should be used as input variables from the viewpoint of contribution to the output (Fujiwara et al., 2012b). The *j*th PLS model with the number of latent variables *P*, $f_{j}^{P}$, is built from the *j*th variable group matrix ***X***_*j*_, and its contribution is evaluated by

(10) $$\begin{array}{r} C_{j}^{P}=1-\frac{||{\hat{y}}_{j}^{P}|{|}^{2}}{||y|{|}^{2}} \end{array}$$

where ${\hat{y}}_{j}^{P}$ is the estimate of $f_{j}^{P}$. We select *D* (≤ *J*) variable groups in descending order of $C_{j}^{P}$ and construct the final PLS model from the selected input variables.

NCSC-GL selects variable groups by using group Lasso instead of contribution evaluation in NCSC-VS. Group Lasso is an extension of Lasso for selecting some input variable groups from predefined multiple variable groups (Yuan and Lin, 2006; Bach, 2008).

Suppose that *M* variables are divided into *J* groups; and ***X***_*j*_ and **β**_*j*_ denote the input data matrix and the regression coefficient vector corresponding to the *j*th group, respectively. The number of variables in the *j*th group is *M*_*j*_, that is, $M=\overset{J}{\underset{j=1}{\sum }}M_{j}$. The regression coefficients of group Lasso is derived as:

(11) $$\begin{array}{r} \beta_{glasso}={\arg\text{ min}}_{\beta }\;\left(||y-\overset{J}{\underset{j=1}{\sum }}X_{j}\beta_{j}|{|}_{2}^{2}+\lambda \overset{J}{\underset{j=1}{\sum }}\sqrt{M_{j}}||\beta_{j}|{|}_{2}\right) \end{array}$$

where $\beta ={\left[\beta_{1}^{T},\cdots \;,\beta_{J}^{T}\right]}^{T}$, and λ is a parameter. Variable groups must be constructed in advance in group Lasso. Thus, NCSC-GL uses variable groups formed by NCSC as the input of group Lasso.

NCSC-VS has four tuning parameters: γ in the NC method, the number of variable groups partitioned by SC, *J*, latent variables in the PLS models for variable group evaluation, *P*, and selected variable groups, *D*. On the other hand, there are three tuning parameters in NCSC-GL: γ in the NC method, the number of variable groups *J* formed by SC and λ in group Lasso. These three or four parameters need to be tuned for appropriate input variable selection. However, their joint optimization is burdensome and time-consuming. For more efficient soft-sensor design, the number of tuning parameters should be reduced.

### 3.4. NCVW

A new input variable weighting method, referred to as NC-based variable weighting (NCVW), is proposed to be used instead of variable selection for conserving labor required for parameter tuning. The proposed method applies the NC method to the input variables and output variable together for calculating similarities based on the correlation between the input variables and output variable, and uses the input variables weighted by the calculated similarities for modeling.

Let the *n*th input sample and the *n*th output sample are $x_{n}\in {ℜ}^{M}$ and *y*_*n*_, where *M* denotes the number of input variables. In NCVW, the NC method is applied to extended samples

(12) $$\begin{array}{r} x_{n}^{\prime }={\left[x_{n}^{\left[1\right]},\cdots \;,x_{n}^{\left[M\right]},y_{n}\right]}^{T}\;\left(n=1,\cdots \;,N\right) \end{array}$$

and the affinity matrix ***S***′ is constructed. Next, the 1st to *M*th element in the (*M* + 1)th column of ***S*** which corresponds to the output variable is extracted as a weighting vector ***w*** = [*w*^[1]^, ⋯ , *w*^[*M*]^]. Finally, a new input variable for PLS modeling is formed as

(13) $$\begin{array}{r} z_{n}=w{}^{\circ}x={\left[w^{\left[1\right]}x^{\left[1\right]},\cdots \;,w^{\left[M\right]}x^{\left[M\right]}\right]}^{T}. \end{array}$$

where ***a***°***b*** denotes an element-wise product between vectors ***a*** and ***b***. Algorithm 3 summarizes the procedure of the proposed NCVW.

**Table:** Algorithm 3 Nearest correlation based variable weighting (NCVW)

1: Prepare ***x***_*n*_ and *y*_*n*_ (*n* = 1, ⋯*N*).
2: $x_{n}\leftarrow {\left[x_{n}^{\left[1\right]},\cdots \;,x_{n}^{\left[M\right]},y_{n}\right]}^{T}\;\left(n=1,\cdots \;,N\right)$
3: Get ***S*** ∈ℜ^(*M*+1) × (*M*+1)^ by applying Algorithm 2 to ***x***_*n*_.
4: Extract the 1st to *M*th element in the *M*+1th column of ***S*** as ***w*** = [*w*^[1]^, ⋯ , *w*^[*M*]^].
5: $z_{n}=w{}^{\circ}x={\left[w^{\left[1\right]}x^{\left[1\right]},\cdots \;,w^{\left[M\right]}x^{\left[M\right]}\right]}^{T}\;\left(n=1,\cdots N\right)$.
6: Construct a model from ***z***_*n*_ by PLS.

In soft-sensor design, the correlation among multiple input variables needs to be considered as well as the correlation between an individual input variable and the output variable. Thus, the proposed NCVW does not evaluate the correlation between each input variable and the output variable, but the correlation of multiple input variables together, which may contribute to an improvement in the estimation performance of a soft-sensor. In addition, the proposed NCVW has only one parameter, which is the threshold of the NC method γ. This leads to a huge efficiency improvement of soft sensor development.

## 4. Case study

This case study evaluates the performance of the proposed NCVW through application to pharmaceutical data provided by Daiichi Sankyo Co., Ltd. (Kim et al., 2011).

### 4.1. Objective data

The objective of this case study is to design a calibration model that estimates active pharmaceutical ingredient (API) content in a target drug. NIR spectra (2203 points in 800−2500 nm) and the API content were measured from the granules of the drug through experiments. Since the number of wavelengths in NIR spectra was large, appropriate input wavelengths of NIR spectra had to be selected for constructing a precise calibration model. The modeling data and validation data consisted of 576 and 20 samples, respectively.

### 4.2. Model construction

Before modeling, a first-order differential Savitzky-Golay smoothing filter (Savitzky and Golay, 1964) was applied to the spectra. As a benchmark, a PLS model using all the wavelengths as the input was constructed, which was called PLS-All. The number of its adopted latent variables was determined by cross-validation. Input wavelengths were selected using PLS-Beta, VIP, stepwise, Lasso, NCSC-VS, and NCSC-GL. Parameters used in each method were selected by trial and error, which are shown in Table 1. We calculated the root-mean-square error (RMSE) for the modeling data in each parameter and determined the optimal wavelengths based on the calculated RMSE.

**Table 1:** Tested parameters.

	**Parameters**
PLS-All	–
PLS-Beta	ν = {0.70, 0.75, 0.80, 0.85, 0.90, 0.95}
VIP	η = {0.6, 0.7, 0.8, 0.9, 1.0, 1.1}
Lasso	λ = {0.1, 0.2, 0.4, 0.5, 0.8, 1.0}
Stepwise	$\bar{p}=\left\{0.005,\;0.05,\;0.08,\;0.1,\;0.12,\;0.15\right\}$
NCSC-VS	γ = 0.99
	*J* = {5, 6, 7, 8, 9, 10}
	*P* = {9, 10, 11}
	*D* = {2, 3}
NCSC-GL	γ = 0.99
	*J* = {5, 6, 7, 8, 9, 10}
	λ = {20, 25}
NCVW	γ = 0.99

We designed PLS models with the wavelengths selected by each method in which cross-validation was used for determining the appropriate number of latent variables. Although Lasso derives regression coefficients, the PLS model was built from the wavelengths whose regression coefficient was not zero. This is for the reason that the number of retained wavelengths was still large and dimension reduction by PLS may have been needed. On the other hand, in the proposed NCVW, we calculated variable weights and constructed the PLS model from the weighted wavelengths. Finally, the API content was estimated by these constructed PLS models.

These procedures were repeated 100 times for calculating average CPU time per one modeling of each method. The computer configuration was as follows: OS: Windows10 (64bit), CPU: Intel Core i7-8700 (3.2 GHz × 6), RAM: 64G bytes, and MATLAB 2018a.

**Figure 2:**
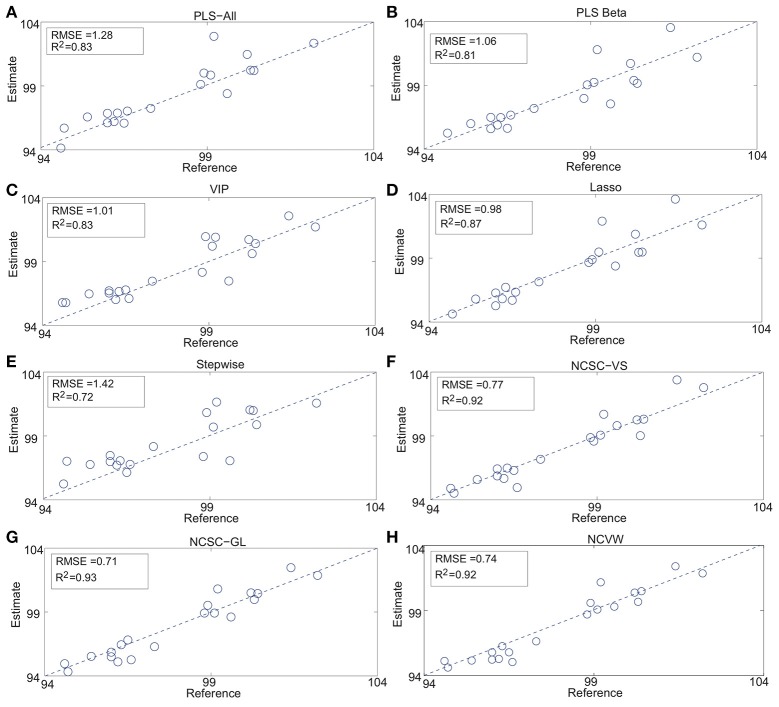
API content estimation results: **(A)** PLS-All, **(B)** PLS Beta, **(C)** VIP, **(D)** Lasso, **(E)** Stepwise, **(F)** NCSC-VS, **(G)** NCSC-GL and, **(H)** NCVW (Fujiwara et al., 2010, 2012a,b).

Table 2 summarizes the results of the case study. #Wavelength and #LV mean the numbers of selected wavelengths and adopted latent variables determined by cross-validation, *R*^2^ is the determination coefficient, “CPU time” is the average CPU times [s], and “Parameters” denotes the optimal parameters in each method. In addition, Figure 2 shows the detailed estimation results.

**Table 2:** API content estimation results.

	**#WL**	**#LV**	**Parameters**	**RMSE**	***R*^2^**	**CPU time [s]**
PLS-All	2203	37	–	1.28	0.83	–
PLS-Beta	928	36	ν = 0.75	1.06	0.81	1.52
VIP	1133	19	η = 0.8	1.01	0.83	0.36
Lasso	1138	39	λ = 0.2	0.98	0.87	0.17
stepwise	561	24	$\bar{p}=0.15$	1.42	0.72	1.64
NCSC-VS	843	25	γ = 0.99, *J* = 6, *P* = 10, *D* = 2	0.77	0.92	202.39
NCSC-GL	1059	18	γ = 0.99, *J* = 8, λ = 25	0.71	0.93	204.04
NCVW	2203	15	γ = 0.99	0.74	0.92	202.27

While PLS-Beta, VIP, and Lasso improved the estimation performance compared to PLS-All, only stepwise was worse than PLS-All. Both NCSC-VS and NCSC-GL achieved higher performance than methods above; and, in particular, NCSC-GL had the best performance. The proposed NCVW achieved almost the same performance as NCSC-VS and NVSC-GL, even though NCVW has only one tuning parameter. RMSE of NCVW was improved by about 42% in comparison with PLS-All.

It is concluded that the proposed NCVW is a tuning-free soft-sensor design technique and that its performance is comparable to the NCSC-based methods.

### 4.3. Discussion

According to Table 2, the CPU time of NCSC-VS, NCSC-GL, and the proposed NCVW were much longer than those of other methods. NCSC occupied more than 99% of their CPU time since it uses iteration for similarity calculation, which means NCVW does not improve the computational load. In addition, the estimation performance of NCVW was not improved in comparison with NCSC-GL; however, construction of the actual soft-sensor therewith is much easier than NCSC-VS and NCSC-GL. The latter methods respectively have four and three tuning parameters. In this case study, 36 calculations in NCSC-VS and 12 calculations in NCSC-GL were repeated for searching the best parameter combination according to Table 1. It becomes difficult to find the optimal parameter combination when the number of tuning parameters increases. On the other hand, NCVW has just one parameter–the threshold of the NC method γ and its recommended value has been proposed to be γ = 0.99 (Fujiwara et al., 2010, 2011). In fact, the total computation times of NCSC-VS, NCSC-GL, and the proposed NCVW were about 121, 42, and 3 min, respectively, for parameter tuning in this case study. Thus, the proposed NCVW makes the soft-sensor design much more efficient than NCSC-VS and NCSC-GL.

Variable weighting based on another type of the weight, the correlation coefficient between each input variable and the output variable, was evaluated. This method is called correlation coefficient-based variable weighting (CCVW). The *m*th variable weight of CCVW is defined as follows:

(14) $$\begin{array}{r} c^{\left[m\right]}=\frac{y^{T}x^{\left[m\right]}}{\left||y||||x^{\left[m\right]}|\right|} \end{array}$$

where ***x***^[*m*]^ ∈ ℜ^*N*^ denotes the *m*th column in the input data matrix ***X*** ∈ ℜ^*N*×*M*^ and ***y*** ∈ ℜ^*N*^ is the output data vector. A PLS model was constructed from the input variables weighted by *c*^[*m*]^. RMSE and *R*^2^ of NCVW were 1.34 and 0.84, respectively. This showed the effectiveness of the variable weight by NCVW which consider the correlation of multiple input variables and the output variable together.

**Figure 3:**
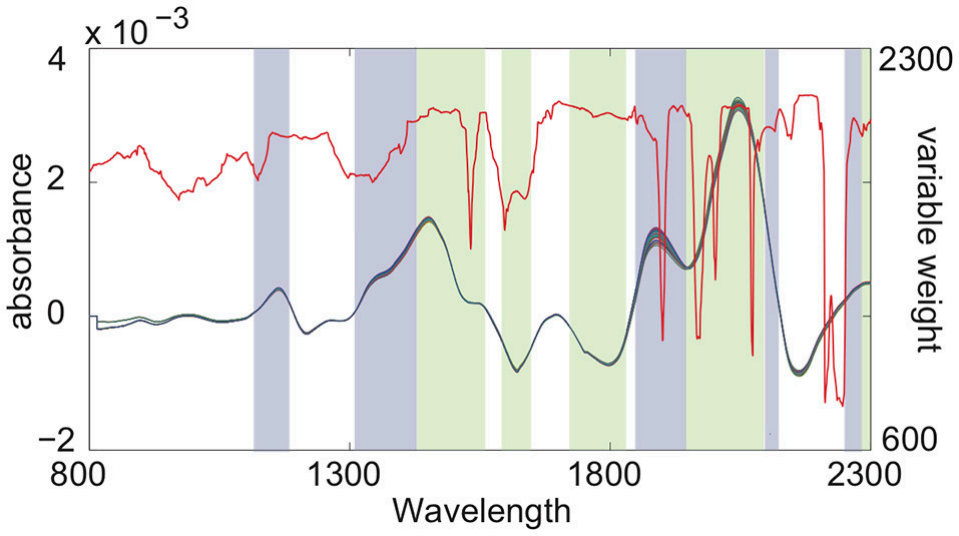
Wavelength group selection by NCSC-VS and variable weights by NCVW.

Figure 3 shows the results of wavelength selection of NCSC-VS and the variable weights calculated by the proposed NCVW. The colored bands express the selected wavelengths, and the colors denote groups by NCSC-VS. The red line is the weights of NCVW. The wavelength groups selected by NCSC-VS contained almost only specific peaks. On the other hand, in NCVW, the weights of almost all wavelength regions that contain peaks, were large while some peaks had small weights. This is consistent with the physicochemical knowledge that information about compounds is contained in specific peaks. Some peaks might have important information about the API content, and other peaks might not contribute to API content estimation. Therefore, the weights by NCVW suggest that unnecessary peaks for API content estimation exist in NIR spectra. This indicates that NCVW can create meaningful weights for soft-sensor design.

## 5. Conclusion

In the present work, an input variable weighting method was proposed for efficient and highly-accurate soft-sensor design. The proposed NCVW derives the variable weights on the basis of the correlation between the input variables and output variable by utilizing the NC method and builds a PLS model from the weighted input variables. Since NCVW has just one tuning parameter, its soft-sensor design is efficient. The performance of NCVW was evaluated through the case study of calibration model development of the pharmaceutical process. The result showed that the estimation performance of NCVW was comparable to that of NCSC-VS and NCSC-GL, while the labor required for parameter tuning was greatly conserved. Although the objective data used in the case study was NIR spectra data, the application area of the proposed method is not limited to a specific type of data. The proposed NCVW is applicable to general soft-sensor design when the number of input variables is large. Therefore, NCVW will contribute to realizing the efficient soft-sensor design.

## Author contributions

KF developed the proposed method, analyzed the data, and wrote the initial draft of the manuscript. MK contributed to data collection and analysis and assisted in the preparation of the manuscript. Both authors approved the final version of the manuscript, and agree to be accountable for all aspects of the work.

### Conflict of interest statement

The authors declare that the research was conducted in the absence of any commercial or financial relationships that could be construed as a potential conflict of interest.
